# Evaluation of time to endosonographic creation route after endoscopic ultrasound-guided hepaticogastrostomy

**DOI:** 10.1055/a-2817-1109

**Published:** 2026-03-16

**Authors:** Yuki Uba, Takeshi Ogura, Saori Ueno, Atsushi Okuda, Nobu Nishioka, Jun Sakamoto, Jun Matsuno, Mitsuki Tomita, Nobuhiro Hattori, Junichi Nakamura, Takafumi Kanadani, Kimi Bessho, Naoto Aoyama, Kouji Kawakami, Ahmad Fikry Aboelezz, Hiroki Nishikawa

**Affiliations:** 1 13010 2nd Department of Internal Medicine, Osaka Medical and Pharmaceutical University, Takatsuki, Japan; 2 38588 2nd Department of Internal Medicine, Osaka Medical and Pharmaceutical University Hospital, Takatsuki, Japan; 3 2nd Department of Internal Medicine, Osaka Medical College, Takatsuki-shi, Japan; 4 68781 Internal medicine, Tanta University, Tanta, Egypt

**Keywords:** Endoscopic ultrasonography, Intervention EUS, Biliary tract

## Abstract

**Background and study aims:**

Recently, antegrade procedures via the endoscopic ultrasound-guided hepaticogastrostomy (EUS-HGS) have been developed as an alternative technique for failed endoscopic retrograde cholangiopancreatography However, time required for formation of endosonography-created route (ESCR) after EUS-HGS is still unknown. To prevent adverse events (AEs) after stent removal, stent removal using the through mesh technique might be useful. The aim of this study was to evaluate time to ESCR formation.

**Patients and methods:**

Consecutive patients who underwent EUS-HGS using self-expandable metal stents (SEMSs) and EUS-HGS stent removal for performing antegrade procedures were retrospectively enrolled. The primary endpoint was evaluation of the rate of ESCR formation. In the present study, EUS-HGS stent removal was attempted at approximately 14 days.

**Results:**

A total of 104 patients were enrolled in this study. EUS-HGS was performed using by partially covered SEMSs (n = 82) or fully covered SEMSs (n = 22). EUS-HGS stent removal was successfully performed in 102 patients (98.1%). Median interval prior to EUS-HGS stent removal in the study subjects was 13 days (range 12–14 days). Among patients who underwent EUS-HGS stent removal, ESCR formation was confirmed in all cases. Mean procedure time was 24.0 minutes. The rate of AEs was 2.9% (3/104)and all AEs were successfully treated conservatively.

**Conclusions:**

In conclusion, ESCR may have been established by a median of 13 days following EUS-HGS using SEMS; however, time to ESCR formation should be evaluated in a future study.

## Introduction

Endoscopic retrograde cholangiopancreatography (ERCP) is the gold standard technique for diagnosis and treatment of biliary diseases, including for biopsy, stent deployment, and stone removal. However, in cases of duodenal obstruction or surgically altered anatomy, ERCP is sometimes challenging. In such cases, endoscopic ultrasound-guided biliary drainage (EUS-BD) is indicated. Among the various EUS-BD procedures, EUS-guided hepaticogastrostomy (HGS) can be performed for patients with inaccessible papilla 1 2 3 4 5 . More recently, antegrade procedures via EUS-HGS have been developed 6 7 8 9 10 . Although single-session antegrade procedures might be feasible, insertion of various devices, such as cholangioscopes or basket catheters, into the biliary tract carry risk of bile peritonitis. Moreover, accidental dislocation of the guidewire is a potential critical complication during antegrade procedures. On the other hand, antegrade procedures might be safe if the endosonography-created route (ESCR) is observed between the hepatic parenchyma and intestinal wall 11 . For easy performance of antegrade procedures, stent removal prior to the antegrade procedure is needed. However, because the time required for formation of the ESCR after EUS-HGS is still unknown, bile leakage can occur if stent removal is performed before the ESCR has completely formed. In such cases, stent removal using the through mesh technique might be useful for overcoming this issue 7 . The aim of this study was to evaluate time to ESCR formation after EUS-HGS and technical feasibility and safety of the through mesh technique.

## Patients and methods

Consecutive patients who underwent EUS-HGS and EUS-HGS stent removal for performing antegrade procedures from March 2017 to December 2024 were eligible for enrollment in this retrospective study. Inclusion criteria were: 1) age > 18 years; 2) EUS-HGS using fully covered self-expandable metal stents (FCSEMSs) (HANAROSTENT Biliary Fully Covered; M.I. Tech, Seoul, Korea) or partially covered SEMS (PSCSEMSs) (Spring Stopper; Taewoong Medical, Seoul, Korea); 3) performance of EUS-HGS stent removal using the through mesh technique; and 4) performance of antegrade procedures via the ESCR. Exclusion criteria were: 1) performance of antegrade procedures through the EUS-HGS stent; 2) EUS-HGS performed using a plastic stent; 3) history of previous EUS-HGS; 4) presence of ascites; and 5) refusal to participate in this study using the opt-out method.

The study was conducted according to the tenets of the Declaration of Helsinki for biomedical research involving human subjects, and all patients provided written informed consent for study participation. A priori approval was given by the Human Research Committee of Osaka Medical and Pharmaceutical University.

### Technical tips for EUS-HGS stent removal using the through mesh technique

**Fig. 1:**
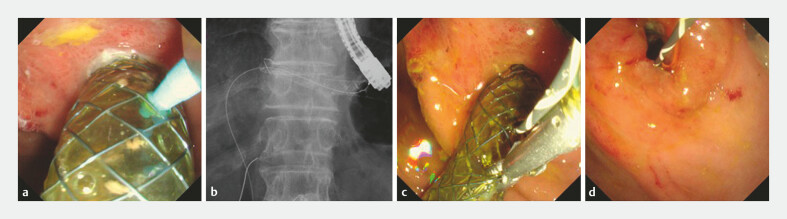
Technical tips for endoscopic ultrasound-guided hepaticogastrostomy (EUS-HGS) stent removal using the through mesh technique. **a** First, the duodenoscope is inserted into the stomach and the ERCP catheter is attached to the mesh of the EUS-HGS stent. **b** A 0.025-inch guidewire is inserted into the biliary tract via the EUS-HGS stent. **c** The EUS-HGS stent is grasped using alligator forceps. d Stent removal is performed through the channel of the duodenoscope.

**Fig. 2:**
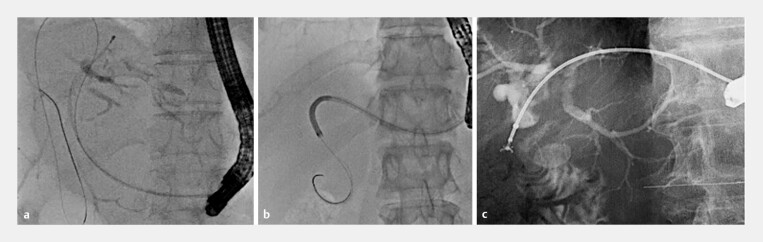
Kinds of antegrade procedures. **a** Stone removal using basket catheter. **b** Cholangioscope insertion using a digital cholangioscope. **c** Antegrade biopsy

Fig. 1 shows technical tips for EUS-HGS stent removal using the through mesh technique. First, the duodenoscope (JF260V, TJF290, Olympus Medical, Tokyo, Japan) was inserted into the stomach and the ERCP catheter (MTW Endoskopie, Düsseldorf, Germany) was attached to the mesh of the EUS-HGS stent ( Fig. 1 **a** ). Then, a 0.025-inch guidewire (VisiGlide 2, Olympus; J-Wire, JMIT, Shiga, Japan) was inserted into the biliary tract via the EUS-HGS stent ( Fig. 1 **b** ) and the ERCP catheter was removed after guidewire deployment. The EUS-HGS stent was grasped using alligator forceps ( Fig. 1 **c** ) and stent removal was performed through the channel of the duodenoscope ( Fig. 1 **d** ). With this procedure, the guidewire was left in to deploy within the biliary tract, and because the ESCR was not formed, reintervention using this guidewire was easily and safely performed. Subsequently, objective antegrade procedures such as stone removal ( Fig. 2 **a** ), cholangioscope insertion using a digital cholangioscope (eyeMAX, Micro-Tech, Nanjing, China; SpyGlass DS II Direct Visualization System; Boston Scientific, Natick, Massachusetts, United States) ( Fig. 2 **b** ), and antegrade biopsy ( Fig. 2 **c** ) were performed.

### Evaluation of ESCR formation

**Fig. 3:**
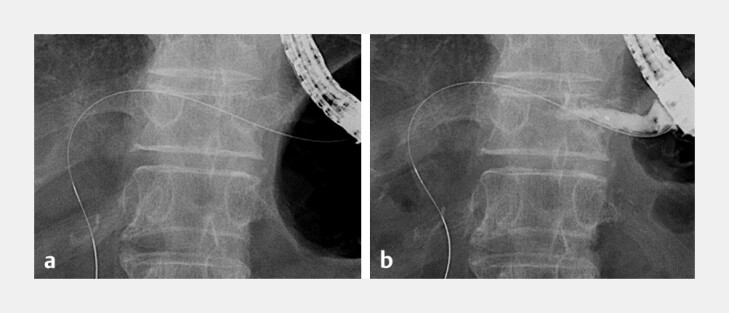
ESCR evaluation. **a** An ERCP catheter is inserted into the ESCR over the guidewire and contrast medium injection is performed. **b** If cholangiography could be performed without contrast medium leakage from the ESCR to the abdominal cavity, it was considered ESCR formation. On the other hand, if leakage of contrast medium from the ESCR or free air were observed, it was considered non-formation of ESCR.

ESCR evaluation was performed after EUS-HGS stent removal using the through mesh technique. After stent removal, an ERCP catheter was inserted into the ESCR over the guidewire ( Fig. 3 **a** ) and contrast medium injection was performed. If cholangiography could be performed without contrast medium leakage from the ESCR to the abdominal cavity ( Fig. 3 **b** ), it was considered as ESCR formation. On the other hand, if leakage of contrast medium from the ESCR or free air were observed it was considered as non-formation of ESCR. Regarding timing of stent removal, we referred to the percutaneous approach. In percutaneous transhepatic access procedures, fistulous tract maturation has been observed to occur predominantly within 2 to 3 weeks when using a transhepatic route, as demonstrated in percutaneous transhepatic gallbladder drainage studies, where most tracts achieved mature sinus formation by 14 days 12 . Therefore, in our center, an HGS stent was removed approximately 14 days later to avoid difficulty with stent removal.

### Definitions and statistical analysis

The primary endpoint of this study was evaluation of the rate of ESCR formation. Adverse events (AEs), the technical success rate of stent removal using the through mesh technique (defined as successful stent removal through the scope without stent breakage or guidewire dislocation), and technical success rates of the trans-ESCR procedures were secondarily evaluated. AEs were graded according to the severity grading system of the American Society for Gastrointestinal Endoscopy lexicon 13 . Bleeding events were defined as bleeding that required blood transfusion, melena, hematemesis, bleeding confirmed on computed tomography (CT), or a decrease in hemoglobin by ≥ 2 g/dL. Technical success of the trans-ESCR procedure was defined if the objective procedure was successfully performed via the ESCR. Mean length of hepatic parenchyma at the puncture site was measured on EUS imaging after successful bile duct puncture. The diameter of the bile duct at the distal end of the EUS-HGS stent was also measured on fluoroscopic images. Mean distance between the hepatic parenchyma and stomach wall and the length of the EUS-HGS stent in the stomach was measured using CT, which was performed the day after EUS-HGS. The time interval prior to stent removal was measured from the day of EUS-HGS to the day of stent removal. Procedure time was also measured from duodenoscope insertion to scope removal. Descriptive statistics are presented as the mean ± standard deviation or median and range for continuous variables, and as frequency for categorical variables. Comparison was performed using analysis of variance for continuous factors, the Kruskal–Wallis test for number of events, and Pearson’s chi-square test or Fisher’s exact test for categorical factors.

## Results

### Patient characteristics

During the study period, antegrade procedures via EUS-HGS were attempted in 136 patients. Among them, 32 patients were excluded from this study due to EUS-HGS being performed with a plastic stent (n = 22), presence of ascites (n = 7), or antegrade procedures being performed through the EUS-HGS stent (n = 3). Finally, a total of 104 patients (median age, 74 years; 62 men) were enrolled in this study. Among them, EUS-HGS was performed using by PCSEMSs (n = 82) or FCSEMS (n = 22) ( Table 1 ). The primary disease was mainly bile duct stones or anastomotic stricture in both groups with no significant differences ( *P* = 0.81). Also, the reason for EUS-HGS was mainly based on surgically altered anatomy. There were no significant differences between the two groups in entry route such as B3 and B2 ( *P* = 0.93). The diameter of the EUS-HGS stent was 8 mm in 61 and 14 patients in the PCSEMS and FCSEMS groups, respectively, and 10 mm in the remaining 21 and 8 patients in the PCSEMS and FCSEMS groups, respectively. Length of the EUS-HGS stent was 12 cm in most cases of both groups. Mean length of hepatic parenchyma at the puncture site was not significantly different between the PCSEMS (20.9 ± 7.12 mm) and FCSEMS groups (20.5 ± 6.70 mm) ( *P* = 0.73). Mean diameter of the bile duct at the distal end of the EUS-HGS stent also was not significantly different between the PCSEMS (3.17 ± 1.50 mm) and FCSEMS groups (3.32 ± 0.78 mm) ( *P* = 0.57). According to CT imaging the day after EUS-HGS, mean distance between the hepatic parenchyma and stomach wall in the PCSEMS and FCSEMS groups was 13.3 ± 7.74 mm, and 12.9 ± 6.96 mm, respectively ( *P* = 0.40) and mean length of the EUS-HGS stent in the stomach in the PCSEMS and FCSEMS groups was 54.8 ± 13.7 mm, and 54.0 ± 11.9 mm, respectively ( *P* = 0.66).

**Table:** **Table 1** Patient characteristics.

	**PCSEMS**	**FCSEMS**	***P* value **
Total number of patients, n	82	22	-
Median age, years (range)	75 (47 – 94)	72 (48 – 92)	0.82
Male:female	40:42	12:10	0.63
Primary disease			0.81
Cholangiocarcinoma	13	2	
Pancreatic cancer	5	1	
Gastric cancer	4	1	
Bile duct stone	26	9	
Anastomotic stricture	31	7	
Others	3	2	
Reason for EUS-HGS			0.61
Surgical altered anatomy	67	19	
Duodenal stenosis	15	3	
Entry route of EUS-HGS			0.93
B2	8	2	
B3	74	20	
Diameter of EUS-HGS stent, n			0.32
8 mm:10 mm	61:21	14:8	
Length of EUS-HGS stent, n			0.93
10 cm:12 cm	7:75	2:20	
Mean length of hepatic parenchyma at puncture site, mm (± SD)	20.9 ± 7.12	20.5 ± 6.70	0.73
Mean diameter of bile duct at distal end of EUS-HGS stent, mm (± SD)	3.17 ± 1.50	3.32 ± 0.78	0.57
Mean distance between hepatic parenchyma and stomach wall, mm (± SD)	13.3 ± 7.74	12.9 ± 6.96	0.40
Mean length of EUS-HGS stent in stomach, mm (± SD)	54.8 ± 13.7	54.0 ± 11.9	0.66
Mean serum albumin, g/dL(± SD)	3.91 ± 1.37	3.78 ± 1.08	0.78
EUS-HGS, endoscopic ultrasound-guided hepaticogastrostomy; FCSEMS, fully covered self-expandable metal stent; PCSEMS, partially covered self-expandable metal stent; SD, standard deviation.			

### Clinical outcomes

Table 2 shows clinical outcomes of this study. The antegrade procedures attempted were stone removal (n = 35), cholangioscope insertion for evaluation of lesions (n = 38), antegrade biopsy under cholangioscopic guidance (n = 28), and antegrade stenting (n = 3). EUS-HGS stent removal was successfully performed in 102 patients (98.1%). Among the two patients in whom the procedure failed, stent removal could not be performed because of mucosal hyperplasia at the uncovered site of the PCSEMS. These patients underwent EUS-HGS within 14 days. Therefore, an FCSEMS was deployed at the hyperplasia site. After 2 days, the stent was successfully removed using the stent-in-stent technique, as previously described 14 . The median interval prior to EUS-HGS stent removal in the study subjects was 13 days (range 12–14 days). Among patients who underwent EUS-HGS stent removal, ESCR formation was confirmed in all patients. The antegrade procedure was successfully performed in 97.2% of patients (101/104) without ESCR dilation for insertion of various devices. The reason for failure of the antegrade procedure in all three patients in whom the procedure failed was incomplete stone removal. These patients underwent scheduled plastic stent exchange. Mean procedure time was 24.0 minutes. In terms of AEs, all AEs, such as cholangitis (n = 2, mild) or pancreatitis (n = 1, mild) were successfully treated conservatively. All AEs were considered to be associated with antegrade procedures.

**Table:** **Table 2** Clinical outcome.

Type of antegrade procedure, n	
Stone treatment	35
Cholangioscope insertion to evaluate lesions	38
Antegrade biopsy under cholangioscopic guidance	28
Antegrade stenting	3
Technical success rate for stent removal, % (n)	98.1 (102/104)
The duration prior stent removal, median (days, range)	13 (12–14)
ESCR creation, % (n)	100 (104/104)
Technical success rate of antegrade procedure, % (n)	97.2 (101/104)
Procedure time (min, mean ± SD)	24.0 ± 11.6
Adverse event, n	
Cholangitis	2
Pancreatitis	1
SD, standard deviation.	

## Discussion

In cases of inaccessible papilla due to surgically altered anatomy, antegrade biliary procedures can be performed using balloon enteroscopy (BE) or an EUS-guided approach. According to a recent meta-analysis including 795 patients (95 with the EUS-guided approach and 700 with BE-ERCP) 9 , the overall technical success rate was similar between both groups (risk ratio [RR] 1.08, 95% confidence interval (CI) 0.84–1.38; *P* = 0.57) and the clinical success rate was also similar (RR 0.95, 95% CI 0.75–1.18; *P* = 0.62). However, the rate of AEs was significantly higher with DB-ERCP compared with the EUS-guided approach (RR 0.95, 95% CI 0.12- 3.15; *P* = 0.006). Iwashita et al compared BE-ERCP and EUS-guided antegrade treatment (AG) for removal of bile duct stones in cases with surgically altered anatomy 15 . Among 119 patients, 23 patients underwent EUS-AG and 96 patients underwent BE-ERCP. The technical success rates of EUS-AG and BE-ERCP were 65.2% (15/23) and 69.8% (67/96), respectively ( *P* = 0.80). Although the rate of AEs was not significantly different (17.4% [4/23] versus 7.3% [7/96]; *P* = 0.22), procedure time was significantly shorter with EUS-AG (mean, 42 minutes versus 64 minutes, *P* < 0.001). These reports suggest that EUS-guided access for biliary disease might be advantageous compared with the enteroscopic approach because of the possibility of lower rates of AEs and shorter procedure times.

Recently, lumen-apposing metal stents (LAMSs) have been used as novel devices during EUS-guided drainage. LAMSs have several advantages compared with conventional SEMS, such as their large diameter or ability for deployment without tract dilation. This allows the endoscope to be passed from the entry site into other organs or the digestive tract. Necrosectomy for walled-off necrosis (WON) is an example of procedures that can be performed through a LAMS. Although further evaluation of the utility of LAMSs for WON is needed, procedure time might be shorter with LAMSs compared with plastic stents because ESCR dilation for endoscope insertion is not required. Recently, EUS-guided transgastric ERCP (EDGE) has been reported as a novel procedure 16 17 . According to a recent meta-analysis regarding EDGE in Roux-en-Y gastric bypass anatomy including 470 patients 18 , the technical success rate was 96% and the clinical success rate was 91%. The pooled rate of all AEs with EDGE was 17%. In addition, technical success and AEs were similar to those with laparoscopic or enteroscopic approaches and procedure time and hospital stay were shorter than with other procedures ( *P* < 0.01). Therefore, if a LAMS is successfully deployed, various transluminal procedures, such as ERCP, can be safely performed prior to ESCR formation. On the other hand, this procedure might be challenging for EUS-AG via the EUS-HGS route. In case of EUS-HGS, because the intrahepatic bile duct is not as dilated compared with the cavity of the WON or the digestive tract lumen, the distal flange might not be completely open. In addition, the distance between the hepatic parenchyma and stomach wall is not so close. This fact might influence stent migration or dislocation into the abdominal cavity if a LAMS is used. Therefore, plastic stents or conventional SEMS should be selected. Yu et al reported single-session EUS-AG for stone removal in 13 patients with surgically altered anatomy 10 . In their report, the guidewire was deployed within the biliary tract after successful puncture of the intrahepatic bile duct, and antegrade balloon dilation of the ampulla of Vater was performed. Subsequently, a retrieval balloon was inserted antegrade and stone removal was performed. Technical success was achieved in all patients and stone removal was successfully performed in 12 patients, although AEs, such as asymptomatic fluid collection or bile leakage, were observed. However, single-session EUS-AG has several disadvantages. First, because there are no adhesions between the hepatic parenchyma and stomach wall, bile leakage can occur continuously until stent deployment during EUS-AG. As a result, bile leakage or peritonitis can occur, as previously reported 10 . Second, if the guidewire is dislocated, reintervention might be challenging because of absence of ESCR. Third, because the tract from the hepatic parenchyma to the stomach wall is not as dilated, various devices, such as cholangioscopes, cannot be used. Therefore, the types of EUS-AG that can be performed might be limited. On the other hand, although a prolonged interval is required for performing the required procedure because we must wait until ESCR formation, EUS-AG after ESCR formation might be safe, and allow various devices to be used.

In the present study using our technique, several significant findings were observed. First, we successfully and safely removed the EUS-HGS stent using the through mesh technique in almost all cases. As noted in the introduction, time to ESCR is still not clear. Therefore, if the ESCR is still not formed after EUS-HGS stent removal, reintervention might be challenging. On the other hand, using the through mesh technique, because the guidewire is still deployed after EUS-HGS stent removal, reintervention can be safely performed using this guidewire. Second, because SEMS was used in the present study, various devices, including cholangioscopes, could be inserted into the biliary tract without ESCR dilation. If EUS-HGS is performed using a plastic stent, because of its smaller diameter compared with SEMS, ESCR dilation might be needed prior to cholangioscope insertion. This could lead to ESCR breakage. Finally, the most novel finding of this study was that mean time to ESCR formation was 13 days. In this study, ESCR was successfully created in all cases.

Our study has several limitations. First, this study was retrospective in nature and there also was no comparison group such as stent removal at different time intervals. Second, our findings might be different for patients who undergo EUS-HGS using plastic stents. In EUS-HGS using SEMSs, the distance between the hepatic parenchyma and stomach wall might be shorter compared with those who undergo plastic stent insertion, because the intrascope channel release technique can be applied as previously described 19 . Third, although this study revealed the time to ESCR, the strength of the ESCR is still unclear. Fourth, because ESCR was successfully created in all cases in the present study, risk factors for non-formation of ESCR could not be evaluated. Therefore, further evaluation is needed to clarify ESCR formation factors.

## Conclusions

In conclusion, ESCR formation might occur within 13 days after EUS-HGS using SEMS; however, time to ESCR formation should be evaluated in a future study.
